# Mastering Sedation and Associated Respiratory Events through Simulation-Based Training: A Randomised Controlled Trial Involving Non-Anaesthesiology Residents

**DOI:** 10.3390/ejihpe14030031

**Published:** 2024-02-23

**Authors:** Jean-Noël Evain, Tran Do, Hakim Harkouk, Pierre Drolet, Roger Perron, Mihai Georgescu, Arnaud Robitaille, Issam Tanoubi

**Affiliations:** Medical Simulation Centre, Centre d’Apprentissage des Attitudes et Habiletés Cliniques (CAAHC), Université de Montréal, Pavillon Roger-Gaudry, 2900, Boulevard Édouard-Montpetit, 8e étage, Local N-805, Montréal, QC H3T 1J4, Canada; tran.hoa.do@umontreal.ca (T.D.); hakim.harkouk@umontreal.ca (H.H.); pierre.drolet@umontreal.ca (P.D.); roger.perron@umontreal.ca (R.P.); leonida-mihai.georgescu@umontreal.ca (M.G.); arnaud.robitaille@umontreal.ca (A.R.); i.tanoubi@umontreal.ca (I.T.)

**Keywords:** simulation-based medical education, procedural sedation, airway management

## Abstract

Non-anaesthetists commonly administer procedural sedation worldwide, posing the risk of respiratory events that can lead to severe complications. This study aimed to evaluate whether simulation-based learning could lead to enhancements in the clinical proficiency of non-anaesthesiology residents in managing sedation and related respiratory complications. Following the evaluation of baseline clinical performance through a pre-test simulation, 34 residents were randomly allocated to either participate in an innovative simulation-based learning module (intervention group) or view a brief self-learning video (control group). After a one-month period, their clinical performance was assessed again in a post-test simulation involving respiratory arrest during procedural sedation. Two independent assessors rated each resident’s performance using video recordings and a scoring tool with scores ranging from 0 to 19/19. The two assessments were averaged for each performance, and the pre- to post-test change was calculated for each resident. While baseline clinical performance was similar, mean (SD) increase in clinical performance was significantly greater in the intervention group than in the control group (+2.4 (1.6) points versus +0.8 (1.3) points, respectively; *p* = 0.002). Our simulation-based learning sedation module resulted in the enhanced management of sedation-related complications compared to baseline and minimal self-learning. Simulation-based medical education may offer an effective approach for equipping non-anaesthesiology residents with essential skills to mitigate risks associated with sedation. (ClinicalTrials.gov identifier: NCT02722226).

## 1. Introduction

Sedation, or monitored anaesthesia care, is a drug-induced depression of consciousness, which most often involves intravenous hypnotics or opioids [1]. It encompasses a continuum from simple anxiolysis through conscious sedation and deep sedation to general anaesthesia. The sedation depth depends on the administered agents’ dosage and blood concentration. Clinicians assess the sedation level by clinical examination: verbal contact, response to stimulation, respiratory rate, and upper airway permeability. Sedation facilitates medical or surgical, diagnostic, or therapeutic procedures, ensuring patient comfort and optimal conditions for the operator [2,3,4]. Procedural sedation is provided extensively throughout the world, often by non-anaesthesiologists. Sedation is, however, not without risk and can lead to severe complications—even death—if carried out under sub-optimal conditions or by inadequately trained professionals. Respiratory dysfunction is the dominant complication of sedation, involving several potentially associated mechanisms: upper airway obstruction, central apnoea or hypopnoea, or the aspiration of gastric contents due to the loss of airway protection reflexes [5].

Studies on sedation outside the anaesthetic field often lack power and suffer from heterogeneous definitions of complications. Thus, it is challenging to identify actionable levers to improve patient safety and reduce the risk of complications [6]. Sedation-related respiratory complications may reflect the inadequate competence of providers, resulting from a lack of knowledge about sedation pharmacology and physiopathology, technical airway management skills, and non-technical crisis resource management skills [6]. The need to standardize sedation provided by non-anaesthesiologists has prompted several anaesthesiology societies to establish standards and guidelines worldwide [7,8,9,10]. Most non-anaesthesiology residents express concern about sedation and thus welcome sedation-focused training programs [11]. However, such specific programs are scarce, and self-learning with online educational content, such as instructional videos, may constitute an alternative solution [12].

Simulation-based medical education involves placing students (or professionals) in artificial clinical situations engineered with varying degrees of realism and providing them with a concurrent or subsequent optimised debriefing [13]. This framework generates activating emotions in participants and fosters safe experiential learning [14]. Healthcare simulation enables reflective practice and metacognition. It is well suited to adult learners, who come in with prior knowledge, representations, and sometimes professional experience [15]. Simulation allows the adage “never the first time on the patient” to be respected and bridges specific gaps in traditional clinical training, such as trainees’ random—and often late—exposure to rare events. The pedagogical impact of simulation-based medical education has been evidenced for all Kirkpatrick levels [16], outperforming traditional clinical medical education [17]. Even if the simulation is time- and human-consuming, its cost efficiency has been repeatedly suggested [18,19]. Simulation seems especially relevant for learning about teamwork and crisis resource management [20]. Simulation, due to its potential efficacy in imparting both technical and non-technical skills, is a pivotal element of the anaesthesiology residency program at the University of Montréal. We hypothesized that simulation training could provide non-anaesthesiology residents with the knowledge and skills necessary to manage sedation and its complications. Following the development of an innovative simulation-based learning module, we conducted a randomized controlled trial to evaluate whether it resulted in improvements in the management of sedation-related complications.

## 2. Materials and Methods

### 2.1. Simulation-Based Learning Sedation Module

Our research team, which includes university hospital anaesthesiologists with routine practice in procedural sedation and simulation-based medical education, first developed the simulation-based learning module tested in this trial. Initially, we conducted an informal needs assessment within the field by interviewing residents enrolled in the radiology, internal medicine, and family medicine programs at the University of Montréal to ascertain the extent of procedural sedation integration into their daily practice. Furthermore, we consulted with their program directors to verify the absence of dedicated training specifically tailored to procedural sedation. Based on the needs identified, the Canadian guidelines on sedation [7], and our experience training anaesthesiology residents, we reached a consensus on six distinct learning objectives (see Box 1).

Box 1 **Learning objective of the simulation-based learning sedation module:**

1. Learn the pharmacology of drugs commonly used for sedation and their antidotes.
2. Assimilate the mandatory safety rules related to sedation.
3. Understand the mechanisms of sedation-related respiratory events.
4. Identify the risk factors for sedation-related respiratory events.
5. Master the technical management of sedation-related respiratory events.
6. Apply the critical resource management principles to sedation-related respiratory events.

**Figure 1:**
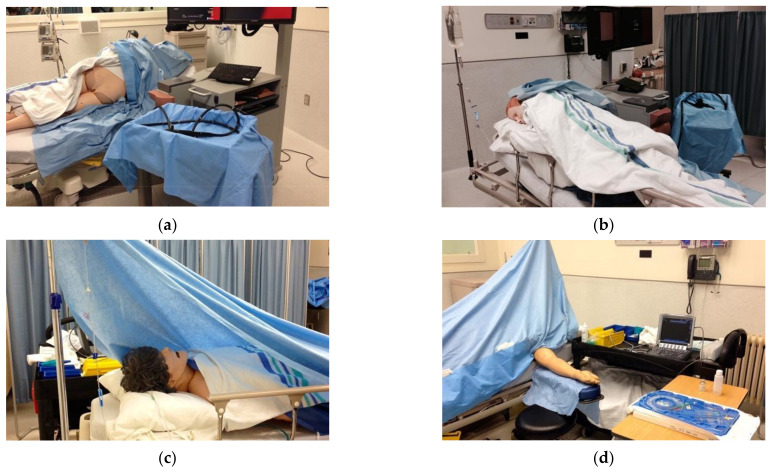
Examples of setups for full-scale, high-fidelity simulation of procedural sedation: colonoscopy (**a**,**b**) and brachial central venous line placement (**c**,**d**).

An integrative workshop, which associated theoretical input and various simulation-based learning modalities, was considered the most appropriate approach for achieving these objectives. We determined the pedagogical content based on the available literature and guidelines, investigators’ clinical expertise, and existing anaesthesiology curricula at the University of Montréal. Three external simulation instructors and anaesthesiologists reviewed the final module. We describe the detailed timeline of the workshop in Table 1. It lasted approximately four hours and comprised four stages. First, participants experienced a full-scale, high-fidelity simulation in which each participant managed the sedation of a patient (SimMan 3G, Laerdal^®^, Stavanger, Norway) who rapidly develops oxygen desaturation. Figure 1 illustrates the simulation setup for two examples of procedural sedation situations. We designed this initial immersive experience to arouse the participants’ curiosity, trigger a constructive discussion, and enable learning objectives to emerge. In the present study, it was also used for pre-test evaluation. The second stage was a theoretical course, where a simulation instructor and anaesthesiologist interactively presented epidemiological, pathophysiological, pharmacological, and therapeutical data on sedation and associated respiratory complications. The third stage was a procedural low-fidelity simulation session with continuous instructor feedback, where the research team invited the participants to practice basic airway management skills (Airway Task Trainers, Laerdal^®^, Stavanger, Norway). Finally, participants underwent a second full-scale high-fidelity simulation where each participant dealt with a respiratory arrest in a patient undergoing procedural sedation. The simulation is interrupted when the trainees establish effective management, and the patient begins to breathe again and wake up. A structured debriefing highlighting the principles of crisis resource management follows and concludes the module.

### 2.2. Setting and Population

After the approval of the local ethics committee (IRB 12-090-CERES-D), this single-blind randomised controlled trial took place at the University of Montréal simulation centre (Centre d’Apprentissage des Attitudes et Habiletés Cliniques, CAAHC, Montréal, QC, Canada). We invited all the residents from the surgery, radiology, pulmonology, and gastroenterology programs at the University of Montréal to participate. Previous specific training in sedation or airway management was an exclusion criterion. We informed each resident of the strict confidentiality of the data collected and the total independence between their participation (or non-participation) in the study and their academic trajectory. Clinical performance was assessed anonymously by assessors external to the resident program. We emphasised kindness and the right to make mistakes as fundamental simulation learning and research principles. Volunteer residents gave their written, informed consent to participate and to be video recorded. The study followed the Consolidated Standards of Reporting Trials guidelines and their extension for simulation research in healthcare [21].

### 2.3. Study Design

**Figure 2:**
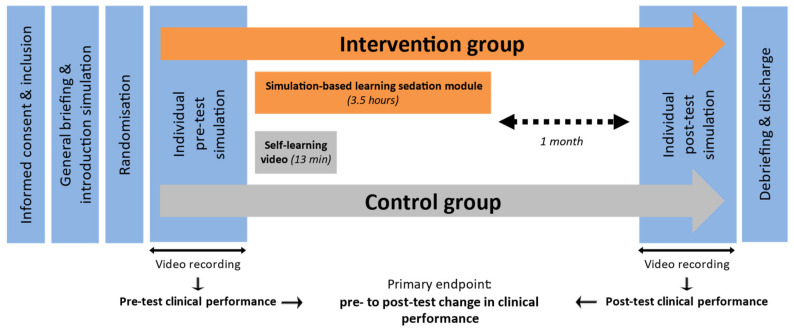
Timeline of the study.

Figure 2 displays the study design. We assessed the pedagogical impact of the simulation-based learning module by comparing the individual clinical performance of trained and untrained residents during a full-scale, high-fidelity simulation of respiratory events in a patient receiving procedural sedation. After inclusion, all participants received a general briefing on the simulated environment and mannequin features and participated in the care of a simulated patient undergoing uncomplicated procedural sedation. Then, they individually performed a pre-test, video-recorded simulation, during which we assessed their baseline clinical performance in managing sedation and associated respiratory events. We adapted the simulated situations (lumbar puncture, bronchial endoscopy, brachial central venous line insertion, colonoscopy) to the resident’s specialty. According to a pre-established computerised randomization table, we assigned each participant to subsequently attend either the rest of the simulation-based learning module (about 3.5 h, intervention group) or to watch a 13 min self-learning video (control group) [12]. After a target period of 1 month, all participants individually performed a post-test simulation, during which the assessors rated their clinical performance again. After the post-test simulation, all participants received a structured debriefing.

Before the study, participants lacked sedation training, despite its probable use in real clinical settings. Notably, those in the control group were offered the module post-study for equitable training. Participation in the study and the sedation module did not affect residency progress.

### 2.4. Primary Outcome Assessment

We designed a full-scale, high-fidelity simulation in which participants’ clinical performance reflected their competency in managing sedation-associated respiratory arrest. The variation in clinical performance between pre-test and post-test simulations would constitute a behaviour change induced by the educational intervention, ranking our trial at the Kirkpatrick level 3. The study scenario was directly adapted from a mature scenario commonly employed within our centre for the training of anaesthesiology residents. We used different scenario variants to mitigate the memory effect. Pre-test and post-test simulations were all videotaped with multi-angle cameras, patient monitor retransmission, and soundtrack from individual and ambient microphones. A facilitator was embedded in the scenario as a respiratory therapist acting neutrally, taking no initiative, and delivering no cues.

Each pre- and post-test clinical performance was assessed by two independent assessors (A and B) blinded to group allocation, using video recordings and a pre-established scoring tool. The scoring tool was a checklist of behaviours expected in the simulated case, developed beforehand through a three-stage modified Delphi survey. First, two investigators from the research team composed an extensive preliminary list of elements based on the workshop learning objectives, available literature, and the specifics of the scenario. Second, five other investigators from the research team revised this initial list by scoring from 1 to 5 the relevance of each proposed element. Third, after erasing items with a median score lower than 3, the edited list was revised once again by five other external experts (also anaesthesiologists from teaching hospitals with routine practice of procedural sedation) with the same scoring protocol. After two rounds of revision, the final checklist comprised 19 items corresponding to expected behaviours, 15 of which had a median relevance score of 4/5 or higher. We divided the 19 items into categories corresponding to the stages of the scenario: initial assessment, diagnosis, management, and secondary assessment. The English translation of the final scoring tool is available in Supplementary Materials (Table S1).

Before the start of inclusions, the checklist designers familiarised assessors A and B with the scoring tool during a specific meeting. We specified the 19 behaviours for calibration purposes and accurately defined their adequate and inadequate execution. Then, based on the video recordings, assessors A and B independently and blindly scored each behaviour as being executed adequately (1 point) or inadequately (0 point), resulting in clinical performance scores ranging from 0 to 19/19. The two assessments were averaged for each performance, and the pre- to post-test change was calculated for each resident.

### 2.5. Statistical Analysis

We conducted statistical analysis utilizing XLSTAT software (version 2023.3.1, Addinsoft, Paris, France). A significance level of *p* ≤ 0.05 was deemed significant, and all tests were two-sided. Quantitative variables were presented as median (Interquartile range, IQR) or mean (standard deviation, SD) based on their distribution, as assessed by the Shapiro–Wilk test. The primary objective of the study was to assess the change in clinical performance from pre-test to post-test. Initially, we aimed to recruit a sample size of 40 residents (20 per group) determined by the estimated population of residents who meet the inclusion criteria at the University of Montréal. With a power of 0.9 and an alpha risk of 0.05, this sample size would enable us to detect a minimum difference of 1 point, assuming a SD of 1 point. To compare the primary endpoint between groups, we employed Student *t*-test. Raw performance scores were compared using the Mann–Whitney test for inter-group comparisons and the Wilcoxon test for intra-group comparisons. Additionally, a post hoc analysis of co-variance was conducted, considering the pre- to post-test delay as a cofactor to explain the primary endpoint.

## 3. Results

### 3.1. Study Population

**Figure 3:**
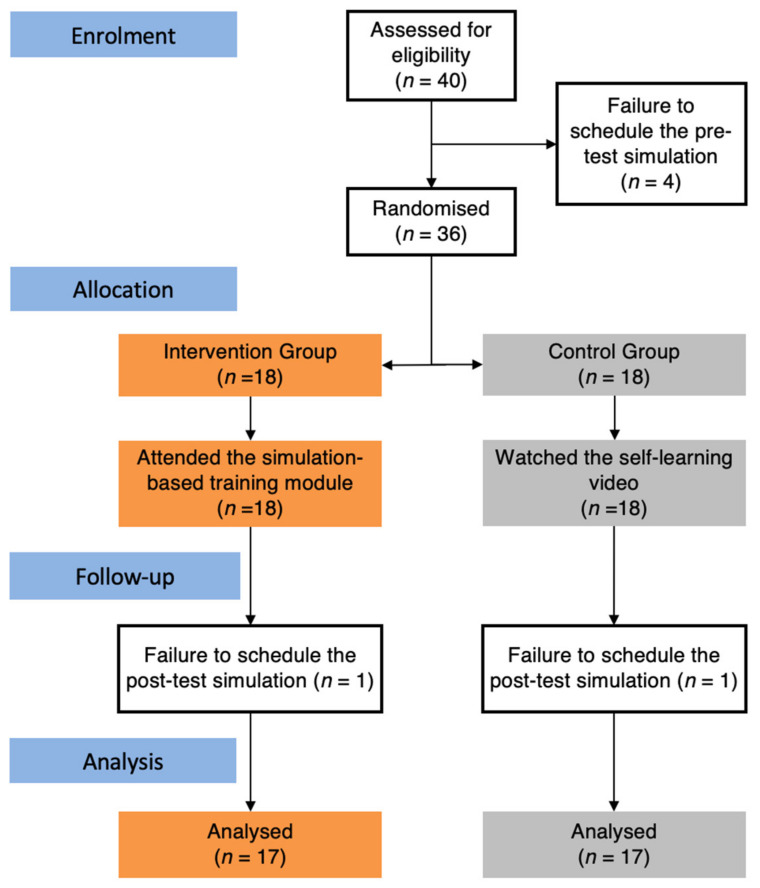
Flowchart of the study.

Figure 3 displays the study flowchart. Of the 40 initial volunteer residents, 36 were randomised and completed the pre-test simulation, and 34 (17 per group) completed the post-test simulation. These residents belonged to different programs (radiology, internal medicine, and family medicine) and varied in seniority (from postgraduate year 1 to 4), with a balanced distribution between the two allocation groups.

### 3.2. Clinical Performance Scores

**Figure 4:**
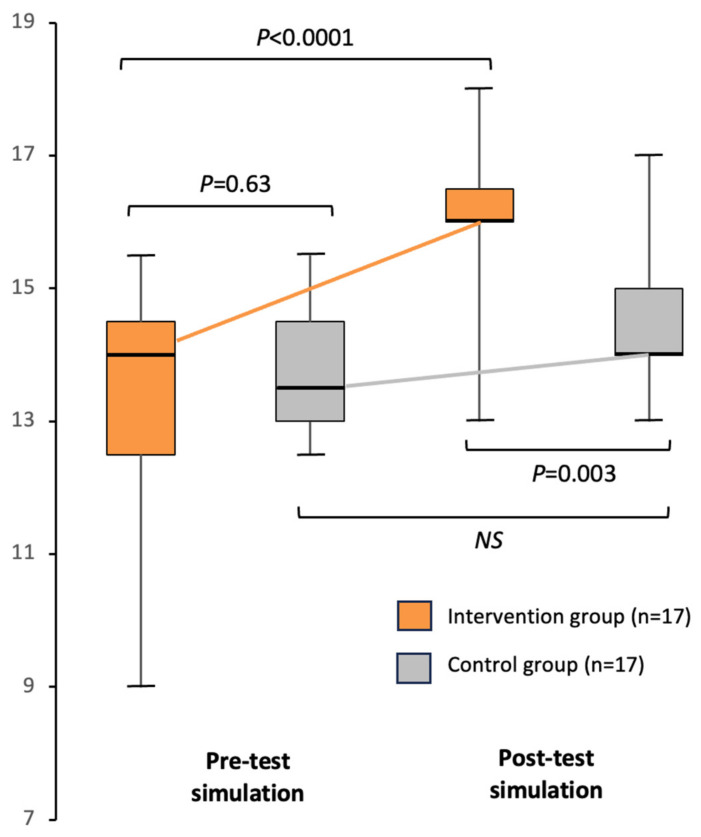
Raw clinical performance scores. Box plots show, from bottom to top, the minimum, first quartile, median, third quartile, and maximum values. NS: not significant.

Figure 4 illustrates the raw clinical performance scores after averaging the two assessments for each performance. The baseline clinical performance (pre-test simulation) was similar between the intervention and control groups (*p* = 0.63). The improvement in clinical performance from pre-test to post-test was significantly greater in the intervention group compared to the control group (mean (SD) = +2.4 (1.6) points versus +0.8 (1.3) points, respectively; *p* = 0.002). Consequently, post-test clinical performance was significantly higher in the intervention group compared to the control group (16.0 [16.0–16.5] versus 14.0 [14.0–15.0] points, respectively; *p* = 0.003).

Clinical performance, as evaluated by assessor A, scored higher than that assessed by assessor B in both pre-test (median [IQR] = 14.0 points [13.3–15.0] versus 13.0 [11.3–14.0], respectively; *p* = 0.002) and post-test simulations (16.5 points [15.0–18.0] versus 14.0 [13.0–15.0], respectively; *p* < 0.0001). The correlation (Pearson coefficient) between the two assessors was significant but weak (ρ = 0.31, *p* = 0.01).

The one-month delay initially targeted proved impossible to meet precisely for most of the participants. The time between pre-test and post-test simulations was variable but similar between the intervention and control groups (median [IQR] = 4 [2,3,4,5] versus 5 [2,3,4,5,6,7,8] weeks, respectively; *p* = 0.55). A covariance analysis revealed that this time delay was not associated with the improvement in clinical performance from pre-test to post-test (*p* = 0.50).

## 4. Discussion

In this randomized controlled trial, non-anaesthesiology residents showed enhanced clinical performance in managing a simulated case of complicated sedation after completing an innovative simulation-based learning module, compared to their performance at baseline or following minimal self-learning from an online educational video.

Several publications highlighted simulation-based education as a high-potential educational approach to learning about sedation in several non-anaesthesiology fields like gastroenterology [22], dentistry [23], or paediatrics [24]. Simulation-based sedation learning facilitates the dissemination of appropriate guidelines to non-anaesthesiologists and may improve their attitude towards the safe management of sedation [25]. Sauter et al. suggested the positive effect of simulation-based education on the management of actual procedural sedation in an emergency department [26]. A literature review revealed, however, that despite the high acceptance of simulation-based education by non-anaesthesiologists practicing sedation, evidence of its pedagogical effectiveness in improving their clinical performance is lacking [27]. Our study is among the first to show that exposure to a simulation-based sedation learning module improves clinical performance in managing a simulated, standardised case of complicated sedation.

The impact of an educational intervention aimed at adults depends on the match between the teachers’ educational objectives, the learners’ expectations, the pedagogical modalities implemented, and the quality of the feedback provided to the learners. Compared to self-learning with no or limited feedback, simulation-based medical education offers a variety of modalities and immersion to optimize the quality of feedback [13]. We combined several modalities to enhance the learning experience in our sedation simulation learning module. First, stimulating learners’ curiosity through a first realistic, full-scale experience of complicated sedation facilitated the transmission of basic knowledge. Then, by providing concurrent and personalised feedback during practice on low-fidelity tasks, trainers facilitated the transfer of technical skills. Finally, the structured debriefing after a second full-scale, immersive experience of complicated sedation facilitated the transfer of non-technical skills.

In this work, we assessed the pedagogical impact of our simulation-based learning module through behavioural changes in response to a simulated clinical situation. Behavioural changes induced by a pedagogical intervention correspond to the third level of Kirkpatrick’s framework [28]. Thus, simulation was used both as a research object [29] and as an evaluation tool [30]. Compared with an authentic clinical situation, measuring these behavioural changes during a simulated clinical situation offers the advantage of both working on a relevant, standardised, and reproducible situation and avoiding any risk to actual patients. Clinical performance evaluation in real-life clinical situations, while theoretically more robust, would have been impossible to carry out in a standardised way. Beyond behavioural change, assessing sedation outcomes in actual patients (the fourth level of Kirkpatrick’s framework) represented a challenge that this work did not set out to meet. Clinical performance remains, however, a problematic construct to evaluate, even in simulation. We advocated using checklists based on the assumptions that clinical performance can be broken down into clearly observable elementary actions [31] and that an observer can accurately assess using checklists. Delphi-type approaches seem to enable the essential matching between checklist contents, the specifics of the simulated case, and best clinical practice [32]. Clinical performance in acute critical situations results from applying technical and non-technical skills. The question of whether to use a global rating scale or a checklist remains incompletely resolved [33]. Global rating scales may be better suited to assessing non-technical skills [34].

This study has several limitations. First, our study does not conclusively establish the superiority of simulation over other potentially relevant educational approaches. Residents in the control group only viewed a brief instructional video, which can be regarded as no intervention. The control group should, therefore, be considered a negative group. Second, the target sample size, based on our estimated inclusion capacity, could not be achieved. Nevertheless, we observed a difference of 1.6 points (out of 19) in performance improvement. Although we are convinced that even modest improvements resulting from educational interventions are significant in the area of patient safety, the clinical relevance of this difference is questionable. A third limitation is that, despite the efforts made to develop a valid grid and to train the assessors, there was a great deal of variation between the assessments. This disparity underscores the inherent challenge of accurately evaluating clinical performance, even within standardized clinical scenarios. Employing the average of two assessments for each performance likely assisted in alleviating this issue. Lastly, the originally planned one-month delay between pre-test and post-test was insufficient for studying long-term skill retention. Given the variable actual delay, we conducted a post hoc covariance analysis to assess its impact on performance change. However, no significant effect was found, leaving the question of skill deterioration over time unresolved.

## 5. Conclusions

Basic knowledge and skills, both technical and non-technical, are essential to mitigate the risks associated with procedural sedation. Simulation-based medical education may offer an effective approach for equipping non-anaesthesiology residents with those knowledge and skills. Following the study results, our sedation module has been incorporated into the standard radiology residency program at the University of Montréal.

## Figures and Tables

**Table 1:** Description of the simulation-based learning sedation module.

	Objective	Pedagogical Approach
Welcome and Simulator Presentation	Foster a positive learning climate Familiarize learners with the simulation environment and mannequin Explain the rules of simulation-based teaching: kindness and respect, confidentiality, and fictional commitment	Interactive discussion and didactic introduction Presentation of the simulation environment and mannequin
Stage 1	Stimulate curiosity and create a learning desire Specify the module’s pedagogical objectives	First immersion in a full-scale, high-fidelity simulation of a complicated sedation case Rapid debriefing of the experience and problems encountered Didactic presentation of learning objectives
Stage 2	Integrate basic knowledge of sedation and associated respiratory complications	Interactive didactic presentation
Stage 3	Transfer basic technical skills in airway management during procedural sedation	Practice procedures on a task trainer Continuous, personalised feedback to optimize gestural behaviour
Stage 4	Apply basic knowledge and technical skills in an immersive, contextualised situation Transfer the non-technical skills required for effective team-based crisis management	Second immersion in a full-scale, high-fidelity simulation of a complicated sedation case Structured debriefing with crisis management analysis
Conclusion and Discharge	Bring out take-home messages	Interactive discussion and didactic presentation

## Data Availability

Study data are available upon request to the corresponding author.

## Supplementary Materials

The following supporting information can be downloaded at: https://www.mdpi.com/article/10.3390/ejihpe14030031/s1, Table S1: Checklist used to rate the clinical performance in managing a simulated case of sedation-associated respiratory arrest (English translation).
