# Supplementary material for: Mastering Sedation and Associated Respiratory Events through Simulation-Based Training: A Randomised Controlled Trial Involving Non-Anaesthesiology Residents
Source: Eur J Investig Health Psychol Educ. 2024 Feb 23;14(3):463–73. doi: 10.3390/ejihpe14030031 (PMC10969482; doi:10.3390/ejihpe14030031)
Supplement: Supplementary file 1 [file ejihpe-14-00031-s001.zip › ejihpe-2846522-supplementary.pdf]

Table S1. Checklist used to rate the clinical performance in managing a simulated case of sedation-associated respiratory arrest (English translation)

INITIAL ASSESSMENT
Checking the patient's medical history
Review of sedative drugs already administered
DIAGNOSIS
Rapid recognition of oxygen desaturation
Rapid recognition of airway obstruction
Verbal stimulation
Pain stimulation
MANAGEMENT (1)
No sedative drugs added
Interruption of the procedure and request for assistance
Increase in oxygen supply
Appropriate patient positioning and cervical extension (chin lift)
Mandibular subluxation (jaw thrust)
Manual ventilation with positive pressure
SECONDARY ASSESSMENT
Checking the saturometer (correctly installed and operational)
Detection of leaks during manual ventilation
Checking the venous access (correctly installed and operational)
Checking the suction (available and operational)
MANAGEMENT (2)
Placement of an oro- or nasopharyngeal cannula
Consideration of the use of an invasive ventilation device
Administration of an appropriate antidote
